# Medication burden attributable to chronic co-morbid conditions in the very old and vulnerable

**DOI:** 10.1371/journal.pone.0196109

**Published:** 2018-04-23

**Authors:** Kelly L. Moore, Kanan Patel, W. John Boscardin, Michael A. Steinman, Christine Ritchie, Janice B. Schwartz

**Affiliations:** 1 Center for Research on Aging of the Jewish Home, San Francisco, CA, United States of America; 2 Department of Medicine, University of California, San Francisco, CA, United States of America; 3 Department of Epidemiology and Biostatistics, University of California, San Francisco, CA, United States of America; 4 San Francisco Veterans Affairs Medical Center, San Francisco, CA, United States of America; 5 Department of Bioengineering and Therapeutic Sciences, University of California, San Francisco, CA, United States of America; Taipei Veterans General Hospital, TAIWAN

## Abstract

**Objective:**

Polypharmacy is common in older patients but relationships between polypharmacy and common co-morbid conditions have not been elucidated. Our goal was to determine relationships between daily oral medication use and common co-morbid disease dyads and triads using comprehensive medication and diagnostic data from a national sample of nursing homes (NH).

**Design:**

Retrospective, cross-sectional study.

**Setting:**

Nationally representative sample of U.S. Nursing Homes.

**Participants:**

Nationally representative sample of long-term stay residents (n = 11734, 75% women) aged 65 years or older.

**Measurements:**

Diagnosis and medication data were analyzed. Proportion of daily oral medication intake attributed to treatment of common two-(dyads) and three-disease (triad) combinations and “health maintenance” agents (vitamins, dietary supplements, stool softeners without related diagnoses) was determined.

**Results:**

Older NH residents received slightly >8 oral medications/day with the number related to number of medical diagnoses (p < .0001). One third of chronic oral medication intake/day (excluding health maintenance agents) could be attributed to dyad combinations and about half to triad combinations despite an average of 5 other diagnoses. Triads were comprised of hypertension +/- arthritis +/- vascular disease, +/-depression, +/- osteoporosis +/- gastroesophageal reflux disease and +/- diabetes. Health maintenance agents accounted for 15–17% of daily oral medication intake (1.4 medications) such that almost two-thirds of daily oral medications were attributable to disease triads plus health maintenance. Fewer medications were prescribed for NH residents over age 85 (decreased ACE inhibitor and HMG CoA reductase inhibitor USE (p < .001)) while use of Alzheimer medications was higher (p < .01).

**Conclusions:**

A large fraction of daily oral medications were attributed to management of common co-morbid disease dyads and triads. Efforts to reduce polypharmacy and unwanted medication interactions could focus on regimens for common co-morbid dyads and triads in varying populations.

## Introduction

Aging is often accompanied by increasing numbers of health-related diagnoses (multi-morbidity) and use of multiple medications (polypharmacy). [1, 2] Surveys estimate that slightly over sixty percent of people over 65 years of age are prescribed three or more medications on a daily basis, and about 39 percent have more than five prescribed. [3–5] The number is even higher in nursing home residents, averaging 6–8 medications per day. [6–14] While the risk of adverse drug events and interactions is directly related to the number of medications consumed [8, 10, 15–22], recommendations to use fewer drugs are often difficult to implement in the face of clinical practice guidelines for multiple medications for each medical condition.

Currently, there are no universally accepted approaches to optimizing medication strategies in patients with multiple co-morbid conditions. Our goal was to investigate use of daily oral medications and daily health maintenance agents in older nursing home residents and determine the relationship between agents administered and the most common co-morbid diseases and comorbid disease combinations. If medication burden could be largely attributed to treatment of a relatively small number of common co-morbid conditions in these complex patients, efforts to improve therapeutic regimens could focus on selection of medications that treat more than one of the underlying conditions to decrease the number of medications and the selection of combinations of medications without predictable adverse interactions.

## Materials and methods

The National Nursing Home Survey (NNHS) methodology has been published elsewhere. [6, 11] In brief, it was a weighted sample from 1,174 facilities and 13,507 residents representing the 1.49 million residents in U.S. nursing homes between August 2004 and January 2005. [23] Our analyses were limited to long-term stay residents older than 65 years. Medication data were from on-site review of medication records—up to 25 medications taken the day before data collection, and up to 15 medications taken regularly but not the day before data collection. [6] Data collection differentiated between as needed medications and those that were prescribed to be taken “regularly” as defined by not having an end-date for administration or on an as needed designation and we considered these as the chronic medications and limited our analyses to those administered orally. Dosage and frequency data and duration of regular or chronic therapy were not provided. Medications were matched to the “Drug Estimates and Characteristics” [24] to obtain drug names and also organized by 2005 National Drug Code (NDC). Administration route was determined by drug labeling information. Data were reduced and coded to reflect active pharmacologic agents/medications in each formulation using standard references (Lexicomp®, Physicians Desk Reference®). Multivitamins, vitamins, nutraceuticals, stool softeners, and bowel stimulants given by mouth daily were considered “daily health maintenance regimen” agents in the absence of a related diagnosis (see below). Non-oral agents, those given for a short course (antibiotics, chemotherapeutic agents) or “as needed” were excluded from analyses.

### Diagnoses data

A maximum of 16 current diagnoses based on ICD-9-CM (International Classification of Disease, 9th Revision, and Clinical Modification) were collected for each resident, with over 99% of residents sampled having fewer than 16. For the primary diagnosis, error estimates were calculated and S.E. ranged from 0.1–0.6. [23]The most frequent chronic medical conditions were identified with Clinical Classifications Software (CCS) for ICD-9-CM developed as part of the Healthcare Cost and Utilization Project (http://www.hcup-us.ahrq.gov/toolssoftware/ccs/ccsfactsheet.jsp). Single level CCS rankings (http://www.hcup-us.ahrq.gov/toolssoftware/ccs/ccs.jsp) were used for initial aggregations of chronic medical conditions identified in over 5% of residents, with additional grouping into single medical conditions based on probable common physiologic basis as previously described. [13]. Example: for “Hypertension”, any ICD-9 code that contained “Hypertension complications” resulted in the resident classified as having hypertension. Gastroesophageal reflux disease heartburn and peptic ulcer disease were combined into a single chronic condition related to acid/peptic acid disease (GERD); and, diabetes with and without complications were combined into a single “diabetes” chronic medical condition. For combined ICD-9 diagnoses of delirium and dementia, delirium diagnoses were excluded to leave only dementia diagnoses. The complete list of ICD-9 codes appearing in the NNHS dataset and corresponding grouped chronic medical conditions are available upon request from the authors[13]. The resulting twenty-one most common chronic medical conditions of atrial fibrillation, anemia, arthritis, atherosclerosis, congestive heart failure, constipation, chronic obstructive pulmonary disease, cerebrovascular disease, dementia, depression, diabetes, GERD /heartburn /ulcer disease, hypertension, lipid disorder, osteoporosis, Parkinson’s disease, peripheral vascular disease, renal failure, thyroid disorders, benign prostatic hyperplasia, and an “all vascular disease” composite diagnosis (including all codes for atherosclerosis, coronary artery disease, cerebrovascular disease, and peripheral vascular disease with these codes not considered individually) were examined in relation to medication usage.

### Medication data

The NNHS dataset does not include medication indications as collected data failed to meet data quality standards for delineating reason for medication use [11]. We took the following approach to assigning potential indications for administration. For formulations with multiple active therapeutic agents (combination products), each active ingredient was considered an individual entity. For individual pharmacologic entities, clinical indications for use were identified by review of 2004 FDA-approved prescribing information and practice guidelines for the 21 chronic medical conditions of interest published up to and including 2005. Multiple indications for an individual agent could exist. For example, indications for metoprolol included hypertension, atherosclerosis or coronary artery disease (including post myocardial infarction), atrial fibrillation, and the composite vascular disease category. Use of vitamins, nutritional supplements, or bowel agents (including laxatives) termed “daily health maintenance agents” were considered therapeutic agents if a diagnosis/clinical indication for use was present. For example, calcium or vitamin D with a diagnosis of osteoporosis or bone fracture, iron or vitamin B12 if a diagnosis of anemia was present, and stool softeners or bowel motility agents with a constipation diagnosis were considered as therapeutic agents. Assignments were reviewed and refined with an expert panel of consultants using a modified Delphi consensus process. Then, each unique medication identified was classified as a) indicated for at least one of the 21 chronic medical conditions, b) indicated for a diagnosis other than the 21 chronic medical conditions considered, or c) a daily health regimen agent. Number of daily oral medications were computed as 1) A+B, and 2) A+B+C.

Estimates of numbers of daily medications for management of co-morbid conditions are presented as the sum of medications potentially indicated for each condition. Estimates of the proportion of daily oral “medication” intake due to daily health regimens were the sum of the number of agents that could not be identified as potentially indicated for a medical condition (as described above). To estimate total daily oral medication intake, health regimen agents were combined with the medications for each disease combination and counted in the total.

### Statistical analysis

Due to the complex probability survey design of the data, SAS survey procedures in SAS version 9.2 (SURVEYMEANS, SURVEYFREQ, SURVEYLOGISTIC) for analyzing survey data were used to account for design effects of stratification and clustering to generate nationally representative estimates. Percentages represent weight- and cluster-adjusted results. Comparisons of characteristics were made between women and men using t-tests for continuous variables using Taylor series method to estimate sampling errors and Rao-Scott Chi-square tests for categorical variables. Standard errors of the prevalence estimates displayed were estimated with the Taylor series linearization method. Corrections for multiple comparisons were made by the Bonferroni method. Linear regression for survey data was used to estimate the relationship between the number of medications and number of diagnoses; and the relationship between age and number of medications administered. Logistic regression was used to estimate odds ratios for age effects as a continuous variable for each sex.

The work was approved for performance as protocol number 12–08640 by the UCSF Committee on Human Research (UCSF Institutional Review Board).

## Results

Data were collected for 11,788 long-term care residents aged ≥ 65 years. Residents without ICD9 data were excluded (n = 54), yielding data on 11,734 residents: 2989 men and 8745 women, representative of 325,919 men and 960,282 women. Table 1 provides demographic and clinical characteristics. The mean age was 84 years with 52% over age 85 years with women on average older than men. (Table 1).

**Table 1:** Characteristics of long-stay nursing home residents aged 65 and older.

Characteristic	All Participants (n = 11734, nw ^a^ = 1,286,201)	Women (n = 8745, nw = 960,282)	Men (n = 2989, nw = 325,919)	Sex Effect
Age (y)	84±0.11	85±0.11	81±0.19	p < .001
65–69	5.1%	37594 (3.9%)	28508 (8.7%)	
70–74	8.0%	59062 (6.2%)	43276 (13.3%)	
75–79	14.3%	118043 (12.3%)	65356 (20.1%)	
80–84	21.3%	202236 (21.1%)	71410 (21.9%)	
85–89	24%	242786 (25.3%)	66036 (20.3%)	
90–94	18.8%	201316 (21.1%)	39898(12.2%)	
≥95	8.6%	99245 (10.3%)	11435 (3.5%)	
Number of Diagnoses				p = 0.6^b^
Age				
65–74	6.3±0.12	6.3±0.14	6.3±0.16	
75–84	6.5±0.09	6.4±0.09	6.5±0.14	
85+	6.4±0.08	6.4±0.08	6.5±0.14	
Number of daily oral medications (including health maintenance agents)*				p<0.001^b^
Age				
65–74	8.6±0.13	9.0±0.18	8.1±0.17	
75–84	8.3±0.09	8.5±0.10	7.9±0.13	
85+	7.7±0.07	7.8±0.07	7.3±0.14	
Number of daily oral medications (excluding health maintenance agents*)				p < .001^b^
Age				
5–74	7.2±0.12	7.5±0.16	6.7±0.17	
75–84	6.9±0.07	7.1±0.09	6.7±0.12	
85+	6.3±0.06	6.4±0.06	6.0±0.12	

Data are mean ± SEM

^a^ nw = weighted frequency.

^b^p-value for sex difference across all ages.

* health maintenance agents defined as vitamins, nutritional supplements, or bowel agents without a diagnosis as clinical indication for use.

**Fig 1:**
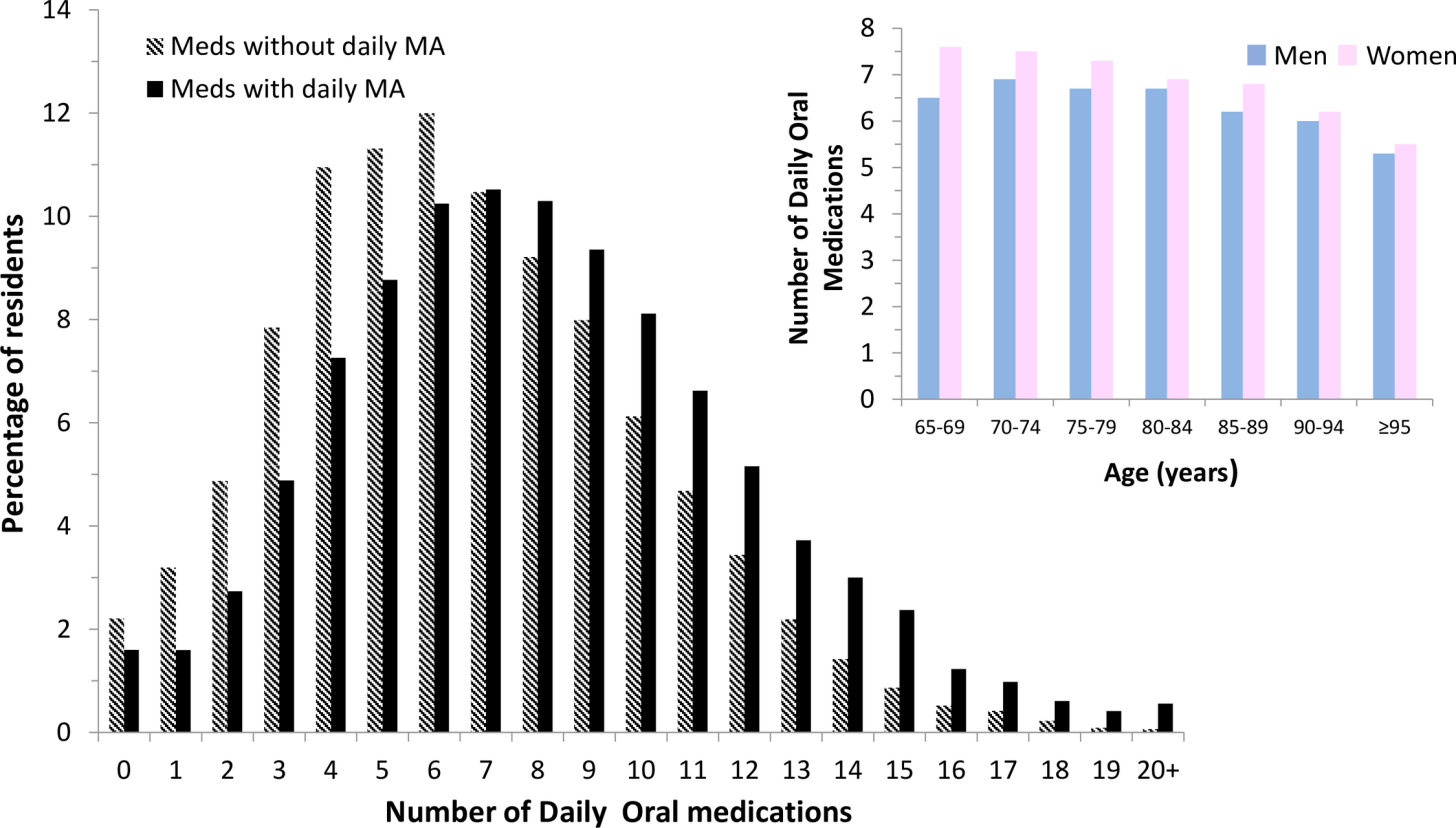
The distribution of the number of chronic daily oral medications for older long-stay nursing homes residents including (solid bars) and excluding (striped bars) daily health maintenance agents (MA) is shown in the larger panel on the left and the mean number of chronic oral daily medications (excluding health maintenance agents) for men (blue) and women (pink) by age group is shown in the smaller inset panel on the right.

Women received slightly more medications than men and mean number of daily oral medications decreased as resident age increased (Table 1, Fig 1) although the number of diagnoses did not differ between women and men or across the agespan of nursing home residents.

Table 2 lists individual medications taken daily in >5% of men or women and effects of age or sex. In men and women, the three most commonly administered medications were aspirin, acetaminophen and furosemide. Age*sex interactions were only detected for atorvastatin (p < .01), carbidopa (p = .007), and L-dopa(p = .004).

**Table 2:** Commonly prescribed chronic oral medications in older long-stay nursing home residents.

Medication	ATC Code#	ATC System/major category	Women (Per cent receiving)	Men (Per cent receiving)	Sex Effect (p)	Age Effect (p)	Odds ratio^ Age Women	Odds ratio^ Age Men
Acetaminophen	N02BE01	Nervous/analgesic	33.8	26.4	0.001	0.006	ns	ns
Aspirin	B01AC06	Blood/antithrombotic	32.7	36.3	0.01	0.87	ns	ns
Furosemide	C03CA01	Cardiovascular/diuretic	29.9	28.0	ns	< .001	1.06*	1.09*
Potassium	A12BA	Alimentary/mineral supplements	24.2	18.7	<0.001	0.03	ns	ns
L-Thyroxine	H03AA01	Hormonal/thyroid	23.7	11.5	<0.001	< .001	ns	1.13*
Lisinopril	C09AA03	Cardiovascular/renin-angiotensin system	14.2	14.1	ns	< .001	0.94*	0.88**
Metoprolol	C07AB02	Cardiovascular/beta blocking	14.2	17.7	<0.001	< .001	ns	0.90*
Donepezil	N06DA02	Nervous-dementia	13.2	13.9	ns	0.71	ns	ns
Citalopram	N06AB04	Nervous/antidepressant	13.1	12.7	ns	< .001	0.91**	ns
Digoxin	C01AA05	Cardiovascular/cardiac therapy	11.3	10.9	ns	< .001	1.14**	1.13*
Mirtazepine	N06AX11	Nervous/antidepressant	10.9	8.7	<0.01	0.001	ns	ns
Amlopdipine	C08CA01	Cardiovascular/calcium channel blocker	10.3	8.9	ns	0.06	ns	0.88*
Warfarin	B01AA03	Blood/antithrombotic	9.7	11.3	ns	0.003	0.92*	ns
Clopidogrel	B01AC04	Blood/antithrombotic	9.7	10.8	ns	0.001	0.92*	ns
Sertraline	N06AB06	Nervous/antidepressant	9.6	9.7	ns	0.12	ns	ns
Ranitidine	A02BA02	Alimentary/acid-related disorders	9.6	10.3	ns	0.64	ns	ns
Pantoprazole	A02BC02	Alimentary/acid-related disorders	9.6	8.6	ns	0.02	0.93*	ns
Lansoprazole	A02BC03	Alimentary/acid-related disorders	8.9	10.3	ns	< .001	0.89**	ns
Hydrochlorothiazide	C03AA03	Cardiovascular-diuretic	8.8	6.9	<0.01	0.22	ns	ns
Olanzapine	N05AH03	Nervous/psycholeptic	8.3	8.6	ns	< .001	0.88**	ns
Quetapine	N05AH04	Nervous/psycholeptic	6.1	8.2	<0.01	< .001	0.91*	ns
Risperidone	N05AX08	Nervous/psycholeptic	7.9	8.1	ns	0.004	0.91*	ns
Celecoxib	MO1AH	Musculoskeletal/anti-inflammatory	7.8	5.9	<0.01	0.39	ns	ns
Atenolol	C07FB03	Cardiovascular/beta-blocking	7.7	6.9	ns	0.89	ns	ns
Omeprazole	A02BC01	Alimentary/acid-related disorders	7.3	7.3	ns	0.93	ns	ns
Isosorbide	C01DA08	Cardiovascular/cardiac therapy	7.2	6.6	ns	0.39	ns	ns
Lorazepam	N05CD06	Nervous/psycholeptic	6.9	5.7	ns	< .001	0.87**	ns
Hydrocodone	N02AA08	Nervous/analgesic	6.9	5.3	<0.01	< .001	0.86**	ns
Atorvastatin	C10AA05	Cardiovascular/lipid-lowering	6.8	8.4	ns	< .001	0.71**	0.81**
Calcitonin	H05BA01	Hormone/calcium homeostatis	5.9	1.8	<0.001	< .001	1.08	1.32*
Gabapentin	N03AX12	Nervous/anti-seizure	5.7	5.7	ns	< .001	0.80**	0.91
Diltiazem	C08DB01	Cardiovascular/calcium channel blocker	5.6	4.6	ns	0.29	0.94	1.01
Paroxetine	N06AB05	Nervous/antidepressant	5.6	4.9	ns	0.82	0.99	0.96
Carbidopa	N04BA02	Nervous/antiparkinson	5.1	8.4	<0.001	< .001	0.81**	0.94
L-Dopa	NO4BA01	Nervous/antiparkinson	4.5	6.9	<0.001	< .001	0.82**	0.96
Risedronate	M05BA07	Musculoskeletal/bone	4.8	1.0	<0.001	0.23	0.98	0.95
Trazodone	N06AX05	Nervous/antidepressant	4.5	4.8	ns	0.12	0.93	1.01
Phenytoin	N03AB02	Nervous/anti-seizure	4.0	6.4	<0.001	< .001	0.76**	0.76**
Memantine	N06DX01	Nervous-dementia	4.0	5.0	ns	0.04	0.93	1.01
Divalproex	N03AG01	Nervous/anti-seizure	3.7	5.8	<0.001	< .001	0.73**	0.83**
Glipizide	A10BB07	Alimentary-diabetes	3.6	5.4	<0.001	< .001	0.8**	0.95

#ATC = Anatomical Therapeutic Chemical Classification system.

^ Odds ratio for age considered as a continuous variable.

* p-value ≤0.01.

** p-value ≤0.001

The number of daily oral medications was positively related to number of diagnoses (p < .0001) without any single chronic disease diagnosis associated with the highest number of daily oral medications. In contrast, residents with dementia were prescribed the lowest average number of daily oral medications (6.1 per day).

We next examined medication use in long stay residents with two co-morbid conditions that were present in at least 10 percent of men or women (Table 3). The dyads included combinations of hypertension, dementia, vascular disease, depression, arthritis, diabetes, GERD, heart failure, anemia, thyroid disease, osteoporosis and chronic obstructive pulmonary disease. Treatment of these dyads explained from 6–30% of daily oral medication intake (excluding the use of daily health maintenance agents) in both men and women. The overall prevalence of the dyads ranged from 7.9–27% and these older residents had an average of more than five additional medical diagnoses.

**Table 3:** Medication usage attributable to common chronic disease dyads.

DYAD	Proportion (%) Daily Oral Medication UseAttributed to Dyad	Total Numberof Daily Oral Medications*	Additional Diagnoses (n)	Overall Prevalence of Dyad (%)
ARTH+HTN	41	7.68 ±0.09	5.5	20
Vasc+HTN	39	7.54 ±0.08	5.3	26.3
Vasc+CHF	36	8.43 ±0.12	5.6	10.4
DEP+HTN	35	7.90 ±0.09	5.5	21
GERD+HTN	35	8.21 ±0.11	5.6	14.2
Vasc+GERD	35	8.18 ±0.13	5.6	11.4
Vasc+DEP	34	8.01 ±0.11	5.5	16.1
Vasc+ARTH	34	7.69 ±0.11	5.5	15
HTN+THYR	34	8.04 ±0.12	5.7	9.7
DIA+HTN	33	8.03 ±0.11	5.6	15.1
ARTH+DEP	33	7.87 ±0.11	5.7	13.5
HTN+OSTEO	32	8.25 ±0.13	5.7	9.6
Vasc+DIA	31	8.14 ±0.13	5.6	12.1
ANEMIA+HTN	31	7.53 ±0.12	5.7	11.5
HTN+DEM	30	6.73 ±0.08	5.3	27.4
Vasc+DEM	28	6.66 ±0.09	5.3	21.1
CHF+HTN	27	8.23 ±0.11	5.7	12.2
ARTH+DEM	26	6.54 ±0.08	5.5	17.4
DEP+GERD	26	8.64 ±0.15	5.8	10.2
COPD+HTN	25	7.76 ±0.14	5.7	7.9
DEM+THYR	23	6.83 ±0.13	5.7	9.2
DEM+DEP	22	6.79 ±0.10	5.5	19.4
DEM+GERD	21	7.23 ±0.13	5.6	10.7
DEM+OSTEO	20	7.08 ±0.13	5.7	9.4
ANEMIA+DEM	15	6.61 ±0.13	5.7	10.3
DEM+DIA	15	7.16 ±0.12	5.6	10.1

Data represent co-morbid 2-disease combinations present in over 5% of either men or women. HTN = hypertension, DEM = dementia, DEP = depression, ARTH = Arthritis, DIA = diabetes mellitus, GERD = gastroesophageal reflux and acid peptic disorders, CHF = congestive heart failure, THYR = thyroid disease, OSTEO = osteoporosis, COPD = chronic obstructive pulmonary disease, Vasc = composite of vascular diseases (atherosclerosis, cerebrovascular disease, coronary artery disease, peripheral artery disease).

*mean ± SEM.

Increasing the number of co-morbid conditions to three increased the proportion of medication use attributable to the three co-morbid conditions to 31–51% of daily oral medication intake (excluding the use of daily health maintenance agents, Table 4).

**Table 4:** Medication usage attributable to common chronic disease triads.

TRIAD	Proportion of Medication Use Attributed to Triad (%)	Number of daily oral medications*	Additional Diagnoses (n)	Overall Prevalence of Triad
HTN+ARTH+DEP	51	8.36 ±0.14	5.2	8.3
HTN+ARTH+OSTEO	50	8.73 ±0.19	5.3	4.0
HTN+ARTH+GERD	50	8.79 ±0.16	5.3	6.2
HTN+Vasc+ARTH	49	8.13 ±0.12	5	9.7
HTN+Vasc+GERD	49	8.51 ±0.15	5.2	7.4
HTN+Vasc+DIA	48	8.36 ±0.15	5	7.9
HTN+Vasc+DEP	48	8.29 ±0.13	5	10.1
HTN+Vasc+THY	47	8.61±0.17	5.1	4.8
HTN+ARTH+DEM	46	7.18 ±0.11	5	10.2
Vasc+DEP+GERD	46	9.03 ±0.20	5.2	5.3
Vasc+ARTH+DEP	46	8.47 ±0.17	5.1	6.4
Vasc+ARTH+GERD	45	8.98 ±0.19	5.2	4.7
HTN+ARTH+ANEMIA	45	8.19 ±0.18	5.3	4.7
HTN+DEP+GERD	45	9.08 ±0.20	5.3	6.5
HTN+Vasc+ANEMIA	44	8.10 ±0.16	5.1	5.6
HTN+Vasc+DEM	44	7.06 ±0.11	4.7	12.6
HTN+ARTH+CHF	42	8.61 ±0.16	5.3	4.7
HTN+DEP+DIA	42	9.01 ±0.18	5.3	5.4
HTN+DEM+DEP	41	7.32 ±0.12	5	11.2
HTN+GERD+DEM	41	7.74 ±0.16	5.1	6.4
HTN+DEP+OSTEO	41	9.06 ±0.20	5.3	4.3
HTN+Vasc+CHF	41	8.66 ±0.15	5.1	6.6
Vasc+DEM+DEP	40	7.32 ±0.14	4.9	8.3
HTN+Vasc+COPD	40	8.16 ±0.20	5.1	4.0
Vasc+DEM+GERD	39	7.57 ±0.17	5	5.2
HTN+DEP+ANEMIA	39	8.32 ±0.19	5.3	4.7
Vasc+ARTH+DEM	39	7.05 ±0.13	4.9	7.5
ARTH+DEM+DEP	38	7.24±0.13	5	7.4
HTN+DEM+DIA	38	7.58±0.15	5.1	6.4
HTN+DEM+OSTEO	37	7.81±0.17	5.1	5.1
HTN+DEM+ANEMIA	37	7.20±0.16	5.1	6.1
Vasc+DEM+DIA	36	7.35±0.17	5.1	5.0
DEM+DEP+GERD	32	7.94±0.19	5.3	5.2
DEM+DEP+OSTEO	31	7.84±0.21	5.3	4.2
HTN+DEM+CHF	31	7.94±0.15	5.1	5.2

Data represent co-morbid 3-disease combinations (Triads) present in over 5% of either men or women. HTN = hypertension, DEM = dementia, DEP = depression, ARTH = Arthritis, DIA = diabetes mellitus, GERD = gastroesophageal reflux and acid peptic disorders, CHF = congestive heart failure, THYR = thyroid disease, OSTEO = osteoporosis, COPD = chronic obstructive pulmonary disease, Vasc = composite of vascular diseases (atherosclerosis, cerebrovascular disease, coronary artery disease, peripheral artery disease).HTN = hypertension, DEM = dementia, DEP = depression, ARTH = Arthritis, DIA = diabetes mellitus, GERD = gastroesophageal reflux and acid peptic disorders, CHF = congestive heart failure, THYR = thyroid disease, OSTEO = osteoporosis, COPD = chronic obstructive pulmonary disease, Vasc = composite of vascular diseases (atherosclerosis, cerebrovascular disease, coronary artery disease, peripheral artery disease).

*mean ± SEM.

Triads were slightly less prevalent than the dyads and were present in 4–13% of the older long-stay nursing home residents. Residents with these triads also had an average of five other diagnoses. Triads explaining the highest proportion of daily medications included hypertension in all, vascular disease or arthritis in half, and depression or GERD in one quarter (Table 4). No sex differences in the proportions of medications attributed to treatment of combinations of the three co-morbid conditions were detected. Health maintenance agents accounted for 15–17% of daily oral medication intake in all residents. Considering the medications attributed to the treatment of the triads combined with oral agents attributable to health maintenance agents thus explained from half to almost two-thirds of daily oral medication intake in patients with the most common three co-morbid conditions studied.

## Discussion

There is current dialogue on how the understanding of multi-morbidity should inform health system design, care guidelines, and quality measures [1, 25]. The impetus for our work was to understand therapeutic regimens in older people with multimorbidity to both simplify regimens and decrease the number of medications prescribed, since polypharmacy is the major risk factor for adverse drug reactions that has been consistently identified. [16, 26–28] We analyzed data from the only survey to date that comprehensively collected individual U.S. nursing home resident medication and diagnostic data from on-site medical record review by trained researchers with methodology procedures to assure accuracy and quality standards. [11, 23]Similar to reports from other countries, polypharmacy was present with about half of the older nursing home residents receiving more than six daily oral medications (excluding health maintenance agents). [29] Thirty-five percent of residents received nine or more oral medications daily. While the number of medications was related to the number of diagnoses, from one-third to half of the oral prescription medication burden of long-term care residents of nursing homes with three co-morbid conditions could be attributed to management of the triad despite the presence of an additional five diagnoses.

Efforts to improve medication usage in older patients and nursing home residents have largely focused on individual high risk medications considered “inappropriate for use in the elderly” [30, 31] or as high-risk in older adults and not based on the prevalence of single or co-morbid conditions. With the exception of off-label use of antipsychotics, medications considered “inappropriate for use in the elderly” during the time of the survey [30, 31] were used infrequently (lorazepam in 6–7%) or not at all (<1%). Subsequent to collection of the data we reported, efforts have reduced use of antipsychotics by 15%, [32] but the challenge of how to optimize medication regimens and minimize adverse events for patients with multiple co-morbid conditions has not been solved. [33,34] Hypertension, vascular disease (coronary, cerebral, or peripheral), dementia, arthritis, depression, and GERD were diagnoses within the most common disease dyads and triads followed by diabetes, heart failure, osteoporosis, and anemia. These data suggest potential starting points for considering drug combinations in relation to common co-morbid diseases with likelihood for polypharmacy.

Some progress has been made in considering medication choices for hypertension in older adults with co-morbid conditions such as coronary artery disease, heart failure, diabetes, and renal insufficiency but not for hypertension in combination with other highly prevalent diagnoses in older adults or those in nursing homes. [35,36] Specifically, antihypertensive treatment guidelines do not consider arthritis, GERD, dementia, depression, osteoporosis or anemia, though these commonly co-exist with hypertension. Nor, do guidelines often consider the duration of therapy needed to see benefits that may be greater than the estimated lifespan of older patients with multiple medical conditions. With a wide range of effective antihypertensive medications from which to choose, consideration of effects on common co-morbid conditions, time to benefit, and the medications to treat co-morbid conditions should be considered if medication therapy is to be optimized. [37] In the NHHS dataset, vascular disease or arthritis were commonly part of three co-morbid conditions that included hypertension followed by depression or GERD. An approach to therapeutic choices to minimize medications could be as follows. For (Hypertension + Vascular Disease), a beta-blocker or calcium channel blocker could treat both conditions and would be preferred to diuretics, ACE, ARB, or renin inhibitors that do not treat atherosclerotic, coronary or cerebrovascular disease. In the presence of arthritis with (Hypertension + Vascular disease), the preferences of agents remain the same. However, if GERD is added to (Hypertension + Vascular Disease), beta-blockers are preferred to calcium channel blockers that may cause or exacerbate GERD. When depression is present, many would avoid beta-blocker use. As the need for medications to treat multiple medical conditions and the choices and potential interactions increase complexity, minimization and deprescribing approaches are less obvious. There is a need for development and testing of approaches, algorithms, and automated real time decision support tools to address the challenge. [4]

Some comments are warranted regarding other patterns of medication use observed. The decrease in number of medications prescribed to the oldest nursing home residents likely represented appropriate attempts to reduce medication burden or side effects near the end of life. Reduced ACE inhibitor use could reflect risks of postural hypotension, hyperkalemia in combination with non-steroidal anti-inflammatory arthritis medications, less stringent blood pressure targets for the elderly with functional impairments, or avoidance of cough side effects. Decreased prescription of HMG CoA-reductase inhibitors likely reflects lesser importance of cholesterol lowering in the very old and mirrors recent international data. [38] Frequency of prescribing did not match frequency of related ICD-9 diagnoses for several medications. Furosemide use was highly prevalent (49%) in residents with a diagnosis of hypertension in the absence of diagnoses of heart failure, edema, or renal disease and thiazide diuretic use was infrequent (<10% of residents). This likely contributed to the use of potassium supplements. Celecoxib was prescribed for 10% or more of patients with triads that included hypertension and may have contributed to the use of multiple medications for treatment of hypertension. Gabapentin use was higher than seizure diagnosis prevalence and was likely used for pain that was not a frequent ICD-9 diagnosis.

A high prevalence of use of health maintaining agents in the absence of a specific diagnosis related to their use was observed and accounted for an average of 1.4 oral medications per day across all age groups. In fact, the number one most frequently prescribed agents were multivitamins in 36% of nursing home residents. Constipation was diagnosed in 10% of residents yet docusate was the fourth most commonly prescribed individual agents in 25% and senna was the ninth most common medication prescribed for 14%. Vitamin C ranked twenty-fourth in daily prescribed medications in 9% of residents. These agents clearly contribute to the polypharmacy and pill burden and may represent classes and categories that should be considered in efforts to reduce polypharmacy.

Our work has limitations. Although 2004 data, the number and demographics of the nursing home population remained unchanged [39] and the diseases and medication prescribing by class have remained stable [40]. Furthermore, prescribing patterns are also very similar to 2013 Medicare Part D claims data, which reports that nine of the top ten medication claims were for treatment of hypertension, cardiovascular disease, GERD, thyroid disorders, and pain and suggests that the patterns noted and opportunities for medication prescribing improvement remain. [41] In both 2004 NNHS and 2013 Medicare claims, lisinopril, L-thyroxine, amlodipine, furosemide and metoprolol were among the ten most commonly prescribed medications. In addition, a distinct pattern of polypharmacy due to vascular disease has also been identified in older outpatients. [42] Data were from clinical care records and billing and diagnostic criteria were not standardized. Medication doses were not reported limiting assessment of inappropriate medication use and only chronic oral medications were considered (e.g. antibiotics, chemotherapeutic agents, “as needed” agents excluded).

In summary, at least half of the medications burden of long-term care residents of nursing homes were attributed to management of three co-morbid diseases. Hypertension, vascular disease, dementia, arthritis, depression, and GERD were diagnoses within the most common three co-morbid conditions. Vascular disease, arthritis, or GERD have not been the focus of efforts to improve medication prescribing in nursing homes. A focus on improving combinations of medications to treat combinations of common co-morbid conditions would be a logical starting point for optimizing care of the elderly nursing home resident with multimorbidity and for those at high risk for adverse drug events but could also be applied to common co-morbid conditions in other settings and patient groups.
